# The value of the cerebrospinal fluid tap test for predicting shunt effectiveness in idiopathic normal pressure hydrocephalus

**DOI:** 10.1186/2045-8118-9-1

**Published:** 2012-01-13

**Authors:** Masatsune Ishikawa, Masaaki Hashimoto, Etsuro Mori, Nobumasa Kuwana, Hiroaki Kazui

**Affiliations:** 1 Normal Pressure Hydrocephalus Center, Otowa Hospital, Kyoto, Japan; 2 Department of Neurosurgery, Noto General Hospital, Nanao, Japan; 3 Behavioral Neurology, Graduate School of Medicine, Tohoku University, Sendai, Japan; 4 Department of Neurosurgery, Tokyo Kyosai Hospital, Tokyo, Japan; 5 Department of Psychiatry, Graduate School of Medicine, Osaka University, Osaka, Japan

## Abstract

**Background:**

The cerebrospinal fluid (CSF) tap test (TT) has been regarded as an important test for the prediction of shunt effectiveness in patients with suspected idiopathic normal pressure hydrocephalus (iNPH). Although its specificity and sensitivity are reportedly high, there remains some disagreement over this point. Herein, the TT as a test for predicting shunt effectiveness was investigated in our multicenter prospective study named SINPHONI and strategies to increase its predictability were examined.

**Methods:**

One hundred suspected iNPH patients with the following entry criteria were enrolled in the study: (1) 60 to 85 years old, (2) one or more of the NPH triad signs, (3) ventriculomegaly (Evans index > 0.3), (4) high convexity tightness in coronal-section MRI, and (5) no antecedent disorders. Changes in NPH triad symptoms were assessed using the iNPH grading scale and other measures before and after removal of 30 ml lumbar CSF. A positive response to TT was pre-defined by specific improvements on the grading and other scales. A ventriculoperitoneal shunt was performed with a programmable valve. The sensitivity and specificity of the TT was calculated with a contingency table. A decision tree analysis was performed to increase the predictability of the TT.

**Results:**

Among 100 patients, 80 were shunt responders. A statistically-significant variable between shunt responders and non-responders was CSF pressure. The changes in single variables in the iNPH grading scale after TT showed high specificity with low sensitivity. In contrast, change of the total score in the iNPH grading scale showed a relatively high sensitivity of 71.3% with specificity of 65%. A decision tree analysis revealed that using the iNPH grading scale total score and pre-shunt CSF pressure ≥ 15 cmH20, sensitivity increased to 82.5%, without a decrease in specificity.

**Conclusions:**

The sensitivity and specificity of the TT for predicting shunt responsiveness were optimum when improvement on any iNPH grading scale was combined with CSF pressure ≥ 15 cmH20. To increase the sensitivity of the TT, further effort is necessary.

**Trial Registration:**

This study is registered with ClinicalTrials.gov, with the number NCT00221091.

## Background

Idiopathic normal pressure hydrocephalus (iNPH) is a cerebrospinal fluid (CSF) shunt-responsive syndrome involving gait disturbance, dementia and urinary incontinence without antecedent disorders, in the elderly. Hakim and Adams first reported improvement of NPH symptoms by removal of 15 ml CSF using a lumbar tap [1]. Wikkelsø *et al*. reported that the tap test (TT) with removal of 40-50 ml CSF was useful for diagnosis and the prediction of shunt response in NPH patients [2]. Since then, there have been a number of studies using removal of CSF volumes via a lumbar tap to predict shunt effectiveness in iNPH patients [3-10]. Because it is easy to perform in neurosurgical and neurological clinics, the Japanese guidelines for management of iNPH recommended TT as an initial invasive test [11,12]. The specificity and sensitivity are reportedly high, but there is some disagreement regarding this between different reports [3-6]. Continuous lumbar drainage for several days with removal of a large CSF volume has been reported to have high sensitivity and specificity [13-17], but it is more invasive for elderly patients that have difficulty in gait, cognition and/or urination. From a clinical standpoint, the effort in performing a TT to increase the predictability of shunt effectiveness is worthwhile, but there has been no prospective validation study in a large number of iNPH patients. In this study, the predictive value of TT was investigated in patients with iNPH using data from a multicenter, prospective study named "Study of idiopathic normal pressure hydrocephalus on neurological improvement; SINPHONI [18]. Special attention was paid to sensitivity and specificity for a number of variables measured before and after the TT.

This study is registered with ClinicalTrials.gov, with the number NCT00221091.

## Materials and methods

### Patients

In 2004, a multicenter, prospective study of idiopathic normal pressure hydrocephalus (SINPHONI) was conducted in Japan [17]. Briefly, it was designed to validate the diagnostic importance of high-convexity tightness in coronal-section MRI [19] with the results of shunt surgery using a programmable valve. The entry criteria were as follows; (1) 60 to 85 years old, (2) one or more of the NPH triad symptoms, (3) ventriculomegaly (Evans Index > 0.3), (4) high-convexity tightness in coronal-section MRI, and (5) no antecedent disorders. The study consisted of one-year registration and one-year follow-up, and was completed in 2006. Data were obtained from 100 patients. The study was a multicenter prospective cohort study conducted in compliance with the Guidelines for Good Clinical Practice and the Declaration of Helsinki (2002) of the World Medical Association. The institutional review board at each site approved the study protocol, and all participants (or their representatives when applicable) gave written informed consent for participation.

### Tap test

A lumbar tap with removal of 30 ml of CSF was performed in all patients. CSF pressure (CSFP) was measured at the site of puncture. Before and after the tap, all patients were evaluated using the iNPH grading scale (GS) [8], the Mini-Mental State Examination (MMSE) and the 3-meter timed up-and-go test (TUG). The iNPHGS is a clinician-rated scale to rate separately the severity of each of the triad symptoms of iNPH **(**disturbances of gait, cognition and urination). The score of each domain ranges from 0 to 4. Grade 0 indicates normal and grade 1 indicates subjective symptoms but no objective disturbance. Grade 2, 3 and 4 indicate mild, moderate and severe disturbances, respectively. The change of gait was evaluated 1 or 2 days after the tap, while change of cognition and urination was evaluated at one week. Assessment was done by neurosurgeons in most cases. Response to the TT was pre-defined by three major scales: iNPHGS, TUG and MMSE. An improvement in one point or more on the iNPH grading scale (each domain and their total), more than 10% improvement in time on TUG, or more than 3 points improvement in the MMSE was regarded as TT-positive. Improvement in any of the total scores of iNPHGS, TUG or MMSE was defined as positive with an additional variable of Tap-any. The sensitivity and specificity of these pre-defined variables as predictors of a response to shunt surgery were calculated. Furthermore, to increase predictability in the responders during clinical practice, a decision tree analysis was applied.

### Shunt surgery

A ventriculo-peritoneal shunt with a Codman-Hakim programmable valve™ (Codman, Johnson and Johnson, Raynham, MA, USA), with the initial pressure setting determined from a quick reference table [20] was installed in all patients within two months after registration. The modified Rankin scale (a scale for measurement of disability) [21] was used as the primary outcome measure, and iNPHGS, TUG and MMSE as secondary outcome measures. Assessment was performed before, and repeated at 3, 6, and 12 months after surgery to determine which patients were shunt responders. A shunt responder was defined as someone who showed an improvement of one point or more on the modified Rankin scale over 12 months.

### Data analysis and statistics

Statistical analysis was performed using JMP statistical software version 9 (SAS Institute, Cary, USA). Statistical comparison was made between shunt responders and non-responders on baseline data, and pre-tap state of iNPHGS, TUG and MMSE (Table 1). Baseline variables include age, Evans index, and CSFP. Pre-tap variables included scores of the three iNPHGS domains (GS-Gait-pre, GS-Cogn-pre, GSs-Urin-pre) and their total scores (GS-Total-pre), MMSE scores (MMSE-pre), and TUG completion times (TUG-pre). These variables were compared between shunt responders and non-responders using chi-squared test. TT-positive patients were counted for each of the variables (GS-Gait-change, GS-Cogn-change, GS-Urin-change, GS-Total-change, MMSE-change and TUG-change), and their sensitivity (%) and specificity (%) were calculated using contingency table. Positive predictive values were not calculated, since they would have been affected by the high prevalence of iNPH in the patient group. Furthermore, a decision tree analysis was performed to determine a practical method for selecting shunt responders with higher sensitivity and specificity. The variables included age, Evans index, CSFP, GS-Total-change, TUG ≥ 10% and MMSE ≥ 3. The former and latter three variables were regarded as continuous and nominal data, respectively. The level of statistical significance was set to *p *< 0.05.

**Table 1:** Baseline characteristics in shunt responders and non-responders measured before tap test.

	Variables	responders (IQR)	non-responders(IQR)	p
1	Number of patients	80	20	

2	Male/Female	44/36	14/6	NS

3	Age (years)*	75(71-78)	75(71.2-78.5)	NS

4	Evans index (%)*	35(33-38)	34.6(32.7-38)	NS

5	CSFP(cmH_2_0)*	13(9-15)	12(8-12.8)	< 0.05

6	GS-Gait-pre*	2(2-3)	2(2-3)	NS

7	GS-Cogn-pre*	2(1-3)	2(1-3.7)	NS

8	GS-Urin-pre*	2(1-3.7)	2.1(1.4)	NS

9	GS-Total-pre*	6(4-9.7)	6(4-9.2)	NS

10	TUG-pre (sec)*	19.5(15-26.7)	20(18.2-27)	NS

11	MMSE-pre*	22.5(15-25.7)	25(18.2-27)	NS

12	SAE (patient numbers)	7	8	< 0.005

	Pneumonia	0	3	
		
	Malignancy	1	1	
		
	Vascular events	3	1	
		
	Subdural effusion	1	0	
		
	Surgery-related**	0	2	
		
	Femoral fracture	2	1	

Abbreviations: IQR: interquartile range, CSFP: cerebrospinal fluid pressure at lumbar tap, GS-Gait-pre: iNPH grading scale for gait pre tap test, GS-Cogn-pre: iNPH grading scale for cognition pre tap test, GS-Urin-pre: iNPH grading scale for urinary function pre tap test, GS-Total-pre: sum of three grading scales, p:probability, TUG-pre: timed up-and-go test, MMSE: Mini-Mental State Examination, SAE: severe adverse events. *: median (25% and 75% IQR),:**: shunt malfunction and bowel injury

## Results

Among the complete patient group, gait disturbance was noted in 91%, cognition disturbance in 80% and urination disturbance in 60%. The number of males vs. females was 58 vs. 42, and median age was 75 (range75-78; 25%IQR-75%IQR) years. Median Evans index was 34.6 (range 32.7-38) and median CSFP at lumbar tap was 12 (range 9-14) cm H2O.

In this study on the diagnostic performance of TT in a total of 100 patients, 80% were shunt responders during the one-year follow-up. Among the 80 shunt responders, improvement of one, two, three or four points on the modified Rankin scale was found in 43, 27, 8 and 2 patients, respectively. Comparison of the preoperative variables between shunt responders and non-responders showed a statistically significant difference for CSFP in that the CSFP was higher in shunt responders, *p *< 0.05 (Table 1). There were no significant differences in Evans index or severity of GS symptoms, TUG or MMSE. The incidence of severe adverse events (SAE) was statistically higher in the non-responders (*p *< 0.005). Among the non-responders, pneumonia was noted in three and surgery-related complications in two (shunt malfunction and bowel injury), while vascular events including cerebral and cardiac infarction in three and femoral fracture in two, occurred among the responders (Table 1).

The sensitivity and specificity for each of the variables were calculated from the number of true positives, true negatives, false positives and false negatives (Table 2). The highest sensitivity was for Tap-any at 92.5%, but its specificity was low at 20%. The highest specificity of 85% was noted on GS-Cogn-change and GS-Urine-change. However, their sensitivity was below 40%. GS-Total-change showed 71.3% sensitivity and 65% specificity. Thus, the sensitivity and specificity changed with different variables and improvement of total score in iNPHGS, which showed sensitivity of 71.3% and specificity of 65%, was most promising among the pre-defined variables.

**Table 2:** Numbers of patients, sensitivity and specificity for each of variables examined.

	Variables	TP	TN	FP	FN	Sensitivity (%)	Specificity (%)
1	GS-Gait-change	41	16	4	39	51.3	80.0

2	GS-Cogn-change	20	17	3	60	25.0	85.0

3	GS-Urin-change	30	17	3	50	37.5	85.0

4	GS-Total-change	57	13	7	23	71.3	65.0

5	TUG ≥ 10% (sec)^1^	26	14	5	50	34.2	73.6

6	MMSE ≥ 3	51	6	14	29	63.8	30.0

7	Tap-any	74	4	16	6	92.5	20.0

8	GS-Total-change, CSFP ≥ 15 cmH_2_O^2^	66	13	7	14	82.5	65.0

Abbreviations: TP: true positives, TN: true negatives, FP: false positives, FN: false negatives (), GS-Gait-change: grading scale gait change 1-2 days after CSF tap, GS-Cogn-change: grading scale cognition change 7 days after tap, GS-Urin-change: grading scale urinary change 7 days after tap, GS-total change: sum of change for all three grading scales, TUG ≥ 10%: ≥ 10% improvement in timed-up -and-go test, MMSE ≥ 3: improvement of 3 or more points on the Mini-Mental State Examination score 7 days after tap, Tap-any: Calculated from improvement in any of the iNPHGS, TUG or MMSE. 1: N = 95 patients, 2: calculated from decision tree analysis.

**Figure 1:**
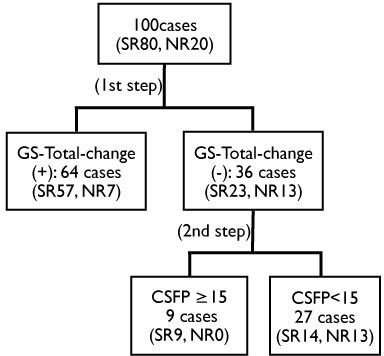
**Decision tree analysis for selecting shunt responders**. At the first step, 57 shunt responders (SR) among 64 patients with improvement of any domain in iNPHGS [GS-Total-change (+)] group were selected as positive cases. At the second step, nine SR were selected from the 36 patients without improvement in iNPHGS [GS-Total-change (-)] group with the variable of CSFP greater than 15 cm H_2_0. This resulted in 82.5% of 80 SR patients being identified in two steps.

To increase predictability of the TT, a decision tree analysis was applied using the variables of age, Evans index, CSFP, GS-Total-change, TUG ≥ 10% and MME ≥ 3 (Figure 1). GS-Total-change was selected as the first node followed by CSFP ≥ 15 cm H_2_O as the second node for differentiating the remaining patients. Using this calculation, the sensitivity was 82.5% and the specificity was 65%.

## Discussion

The response to a lumbar tap test (TT) is considered to be useful for predicting a favourable response to shunt surgery, particularly in iNPH patients. In previous studies, the volume of CSF removed has varied from 30 ml [6,8], 40 ml [4,7], to 50 ml [2], or until pressure was lowered to zero [5]. In the present study, 30 ml CSF was selected because it was less invasive for the elderly patients. One of purposes in the SINPHONI study was to clarify the sensitivity and specificity of the removal of 30 ml CSF for predicting the response to shunt surgery. The present study was designed to detect the change of symptoms as efficiently as possible, after one or two days after the TT for gait and after one week for cognition and urination. Improvement of gait after removal of CSF, was most commonly seen and it could be observed within one or two days after the tap. Recently, Virhammar *et al*. recommended assessment of gait within 24 hours [10]. Improvement of cognition and/or urination is usually more delayed, which was experienced through our preliminary studies including the report by Kubo *et al*. [8]. One disadvantage of the study design was that assessment of iNPHGS, TUG and MMSE was not performed by the same person throughout. This may have caused some inconsistency in the results. This is in contrast to the report by Kubo *et al*. [8]. The MMSE alone would not have been adequate to assess the response to TT. However, it is popular for assessment of cognition in general. Examining the prognostic value of the MMSE was one of objectives in the SINPHONI study.

The sensitivity and specificity of the TT have been reported previously as ranging from 72% to 100% for the former and from 33% to 100% for the latter [3,5,7,8]. The specificity of the TT was reported to be high with low sensitivity [3,7,8], but another report was contradictory [5]. In the present study, the specificity of gait domain was 80% but sensitivity was 51.3%. The cognition and urination domains showed a specificity of 85% in both, but a low sensitivity of 25% and 37.5%, respectively. Thus, the present study revealed a high specificity with low sensitivity in each domain of iNPHGS, which agrees with previous reports [3,7,8]. In contrast to each domain of the iNPHGS, the total GS score showed higher sensitivity of 71.3% but lower specificity of 65%. Among pre-defined variables, the calculated variable of Tap-any showed the highest sensitivity of 92.5%, but the specificity was only 20%. Thus, the sensitivity and specificity of the TT depended on the variable under consideration. In clinical practice, a higher sensitivity would be more preferable for a diagnostic test, although higher specificity is also important to reduce the false positive cases. To increase, both sensitivity and specificity, a decision tree analysis was applied in the present study, which revealed a first node of GS-Total-change. Among the remaining patients, CSF pressure at 15 cm H_2_O was the best threshold for differentiation. This increased the sensitivity to 82.5%, while the specificity remained at 65%. This suggested that patients with higher CSF pressure would be shunt responders even if their symptoms did not improve by one point or more in the iNPHGS after TT.

In contrast with TT, continuous CSF drainage has been reported to provide higher sensitivity and specificity, ranging from 50% to 100% and from 60% to 100%, respectively [7,14-16]. As Marmarou stated, the advantage of continuous CSF drainage is increased sensitivity [13]. Drainage of a larger CSF volume simulates a closer intracranial situation to that following CSF shunt surgery. However, it must be highlighted that most studies involving larger volume drainage, defined shunt responders with symptomatic improvement [7,14-16], not with improvement of daily life activity. In SINPHONI, shunt responders on the iNPHGS, i.e., symptom-basis, were 89% in contrast with 80% on the modified Rankin scale, i.e., function-basis [18]. Thus, caution is needed when comparing the present data with those obtained after larger volume drainage. Although complications were reportedly very low in larger volume drainage [14,15,19], there is potentially more risk for complications in patients who are elderly with a greater or lesser degree of disturbances in gait, cognition, and/or urination.

The SINPHONI study revealed high achievement in the treatment of iNPH patients without support of the TT [18]. The SINPHONI study showed the high predictability and diagnostic importance of MRI features of tight high convexity and enlarged Sylvian fissure with ventricular dilatation, which was designated as "Disproportionately Enlarged Subarachnoid-space Hydrocephalus (DESH)" [18]. However, Iseki *et al*. reported there were asymptomatic people with MRI features of iNPH in their population-based study [22]. They may have been potential candidates for developing iNPH in the future. Because NPH symptoms are often difficult to differentiate from those of other senile disorders, it is important to see the changes of symptoms after the TT or larger volume drainage. To increase the sensitivity of the TT, further effort is necessary.

## Conclusions

The value of the TT for predicting shunt effectiveness was investigated in iNPH patients using the SINPHONI data. The sensitivity and specificity changed with different variables and improvement in any iNPH grading scale showed a sensitivity of 71.3% and specificity of 65%. A decision tree analysis revealed that any improvement on iNPHGS followed by inclusion of patients with CSFP higher than 15 cm H_2_0 increased the sensitivity up to 82.5% without a decrease in specificity. Thus, the TT is valid as an initial invasive test to predict the response to shunt for elderly patients having disturbances of gait, cognition and/or urination.

## List of abbreviations

Cogn: cognition; FN: false negative; FP: false positive; GS: iNPH grading; scale; iNPH: idiopathic normal pressure hydrocephalus; IQR: interquartile range; MMSE: Mini-Mental State Examination; SINPHONI: Study of idiopathic normal pressure hydrocephalus on neurological improvement; TN: true negative; TP: true positive; TT: tap test; TUG: timed up and go test; Urin: urination.

## Competing interests

This investigator-initiated study was supported in part by Johnson & Johnson K.K., Nihon Medi-Physics Co. Ltd, and Daiichi Pharmaceuticals Co. The funding sources for the study had no role in the design and conduct of the study, in the collection, analysis, and interpretation of the data, or in the preparation, review, or, approval of the manuscript. Drs. Ishikawa, Hashimoto, and Mori have received honoraria from companies that manufactured the devices discussed in this article, including Johnson & Johnson K.K. (Japan) and Nihon Medi-Physics Co. Ltd. Drs. Kuwana and Kazui have received honoraria from Johnson & Johnson K.K. (Japan).

## Authors' contributions

All authors had full access to all of the data in the study and take responsibility for the integrity of the data and the accuracy of the data analysis. MI, MH and EM: drafting of the manuscript. NK and HK: critical revision of the manuscript for important intellectual content. All authors have read and approved the final version of the manuscript.

## References

[B1] Hakim S Adams RD The special clinical problems of symptomatic hydrocephalus with normal cerebrospinal fluid pressure: Observations on cerebrospinal fluid dynamics J Neurol Sci 1965 2 302 327 10.1016/0022-510x(65)90016-x 5889177

[B2] Wikkelsø C Andersson H Blomstrand C Lindqvist G The clincial effect of lumbar puncture in normal pressure hydrocephalus J Neurol Neurosurg Psychiatry 1982 45 64 69 10.1136/jnnp.45.1.64 7062072 PMC491267

[B3] Haan J Thomeer RT Predictive value of temporary external lumbar drainage in normal pressure hydrocephalus Neurosurgery 1988 22 388 391 10.1227/00006123-198802000-00020 3352890

[B4] Sand T Bovim G Grimse R Myhr G Helde G Cappelen J Idiopathic normal pressure hydrocephalus: the CSF tap-test may predict the clinical response to shunting Acta Neurol Scand 1994 89 311 316 8085427 10.1111/j.1600-0404.1994.tb02640.x

[B5] Malm J The predictive value of cerebrospinal fluid dynamic tests in patients with th idiopathic adult hydrocephalus syndrome Arch Neurol 1995 52 783 789 10.1001/archneur.1995.00540320059013 7639630

[B6] Stolze H Kuhtz-Buschbeck JP Drucke H Johnk K Diercks C Palmie S Mehdorn HM Illert M Deuschl G Gait analysis in idiopathic normal pressure hydrocephalus - which parameters respond to the CSF tap test? Clin Neurophysiol 2000 111 1678 1686 10.1016/S1388-2457(00)00362-X 10964082

[B7] Walchenbach R Geiger E Thomeer RT Vanneste JA The value of temporary external lumbar CSF drainage in predicting the outcome of shunting on normal pressure hydrocephalus J Neurol Neurosurg Psychiatry 2002 72 503 506 11909911 10.1136/jnnp.72.4.503 PMC1737811

[B8] Kubo Y Kazui H Yoshida T Kito Y Kimura N Tokunaga H Ogino A Miyake H Ishikawa M Takeda M Validation of grading scale for evaluating symptoms of idiopathic normal-pressure hydrocephalus Dement Geriatr Cogn Disord 2008 25 37 45 10.1159/000111149 18025828

[B9] Ishikawa M Oowaki H Matsumoto A Suzuki T Furuse M Nishida N Clinical significance of cerebrospinal fluid tap test and magnetic resonance imaging/computed tomography findings of tight high convexity in patients with possible idiopathic normal pressure hydrocephalus Neurol Med Chir (Tokyo) 2010 50 119 123 10.2176/nmc.50.119 20185875

[B10] Virhammar J Cesarini KG Laurell K The CSF tap test in normal pressure hydrocephalus: evalation time, reliability and the influence of pain Eur J Neurol 2011 doi: 10.1111/j. 1468-1331.2011.03486.x 10.1111/j.1468-1331.2011.03486.x 21801282

[B11] Guideline Committee Japanese Society of Normal Pressure Hydrocephalus Clinical guidelines for idiopathic normal pressure hydrocephalus Osaka: Medical Review 2004 [in *Japanese*]

[B12] Ishikawa M Hashimoto M Kuwana N Mori E Miyake H Wachi A Takeuchi T Kazui H Koyama H Guidelines for management of idiopathic normal pressure hydrocephalus Neurol Med Chir (Tokyo) 2008 48 S1 23 10.2176/nmc.48.S1 18408356

[B13] Marmarou A Bergsneider M Klinge P Relkin N Black PM The value of supplemental prognostic test for the preoperative assessment of idopathic normal-pressure hydrocephalus Neurosurgery 2005 57 S2-17 S2-28 10.1227/01.neu.0000168184.01002.60 16160426

[B14] Marmarou A Young HF Aygok GA Sawauchi S Tsuji O Yamamoto T Dunbar J Diagnosis and management of idiopathic normal-pressure hydrocephalus: a prospective study in 151 patients J Neurosurg 2005 102 987 997 10.3171/jns.2005.102.6.0987 16028756

[B15] McGirt MJ Woodworth G Coon AL Thomas G Williams MA Rigamonti D Diagnosis, treatment, and analysis of long-term outcomes in idiopathic normal-pressure hydrocephalus Neurosurgery 2005 57 699 705 16239882 10.1093/neurosurgery/57.4.699

[B16] Panagiotopoulos V Konstantinou D Kalogeropoulos A Maraziotis T The predictive value of external continuous lumbar drainage with cerebrospinal fluid outflow controlled by medium pressure valve in normal pressure hydrocephalus Acta Neurochir (Wien) 2005 147 953 958 10.1007/s00701-005-0580-9 16041469

[B17] Woodworth GF McGirt MJ Williams MA Rigamonti D Cerebrospinal fluid drainage and dynamics in the diagnosis of normal pressure hydrocephalus Neurosurgery 2009 64 919 925 10.1227/01.NEU.0000341902.44760.10 19404152

[B18] Hashimoto M Ishikawa M Mori E Kuwana N for the study of idiopathic noramal pressure hydroephalus on neurological improvement (SINPHONI) group Diagnosis of idiopathic normal pressure hydrocephalus is supported by MRI-based sheme: a prospective study Cerebrospinal Fluid Res 2010 7 18 10.1186/1743-8454-7-S1-S18 21040519 PMC2987762

[B19] Kitagaki H Mori E Ishii K Yamaji S Hirono N Imamura T CSF spaces in idiopathic normal pressure hydrocephalus: morphology and volumetry AJNR Am J Neuroradiol 1998 9 1277 1284 PMC8332236 9726467

[B20] Miyake H Kajimoto Y Tsuji M Ukita T Tucker A Ohmura T Development of a quick reference table for setting programmable pressure valves in patients with idiopathic normal pressure hydrocephalus Neurol Med Chir (Tokyo) 2008 8 427 432 10.2176/nmc.48.427 18948675

[B21] van Swieten JC Koudstaal PJ Visser MC Schouten HJ van Gijn J Interobserver agreement for the assessment of handicap in stroke patients Stroke 1988 19 604 607 10.1161/01.STR.19.5.604 3363593

[B22] Iseki C Kawanami T Nagasawa H Wada M Koyama S Kikuchi K Arakawa S Kurita K Daimon M Mori E Kato T Asymptomatic ventriculomegaly with features of idiopathic normal pressure hydrocephalus on MRI (AVIM) in the elderly: a prospective study in a Japanese population J Neurol Sci 2009 277 54 57 10.1016/j.jns.2008.10.004 18990411

